# Diet in Irritable Bowel Syndrome (IBS): Interaction with Gut Microbiota and Gut Hormones

**DOI:** 10.3390/nu11081824

**Published:** 2019-08-07

**Authors:** Magdy El-Salhy, Jan Gunnar Hatlebakk, Trygve Hausken

**Affiliations:** 1 Section for Gastroenterology, Department of Medicine, Stord Hospital, 5409 Stord, Norway; 2 Department of Clinical Medicine, University of Bergen, 5007 Bergen, Norway; 3 National Centre for Functional Gastrointestinal Disorders, 5021 Bergen, Norway

**Keywords:** diet, fecal microbiota transplantation, gut endocrine cells, gut microbiota, IBS

## Abstract

Diet plays an important role not only in the pathophysiology of irritable bowel syndrome (IBS), but also as a tool that improves symptoms and quality of life. The effects of diet seem to be a result of an interaction with the gut bacteria and the gut endocrine cells. The density of gut endocrine cells is low in IBS patients, and it is believed that this abnormality is the direct cause of the symptoms seen in IBS patients. The low density of gut endocrine cells is probably caused by a low number of stem cells and low differentiation progeny toward endocrine cells. A low fermentable oligo-, di-, monosaccharide, and polyol (FODMAP) diet and fecal microbiota transplantation (FMT) restore the gut endocrine cells to the level of healthy subjects. It has been suggested that our diet acts as a prebiotic that favors the growth of a certain types of bacteria. Diet also acts as a substrate for gut bacteria fermentation, which results in several by-products. These by-products might act on the stem cells in such a way that the gut stem cells decrease, and consequently, endocrine cell numbers decrease. Changing to a low-FODMAP diet or changing the gut bacteria through FMT improves IBS symptoms and restores the density of endocrine cells.

## 1. Introduction

It has been estimated that 12.1% of the world’s population suffers from irritable bowel syndrome (IBS) [1,2]. The main symptom of IBS is recurrent abdominal pain associated with a change in the bowel habits according to Rome IV criteria [3]. The diagnosis of IBS is based mainly on an assessment of the symptoms as described by Rome criteria [4]. Based on stool patterns, IBS patients are divided into four subtypes: diarrhea-predominant (IBS-D), constipation-predominant (IBS-C), mixed diarrhea and constipation (IBS-M), and patients who meet the diagnostic criteria for IBS, but whose bowel habits cannot be accurately categorized (IBS-U) [5,6].

IBS is usually diagnosed in young patients in a phase of their lives when they are trying to build a family and getting an education/working [7,8,9,10,11,12,13,14,15,16,17,18]. IBS reduces the patient’s quality of life to the same degree as other chronic diseases such as diabetes, inflammatory bowel diseases, and kidney failure [18,19,20,21]. IBS patients generate a substantial workload in both primary and secondary care, and 12–14% of primary care patient visits and 28% of referrals to gastroenterologists are IBS patients [12,13,22]. Thus, IBS is a more common reason for a visit to a physician than diabetes, hypertension, or asthma [23,24].

IBS patients can be divided into two subsets: sporadic (nonspecific) and post-infectious (PI) [19]. Sporadic IBS includes patients who have had symptoms for a long time without any associated events. PI-IBS occurs in otherwise healthy subjects as a sudden onset of IBS symptoms after gastroenteritis [19]. PI-IBS comprises about 6–17% of patients with IBS [25].

There is no effective treatment for IBS, and the treatment practiced in the clinic is directed toward the relief of symptoms [26]. An international survey showed that these patients would give up 25% of their remaining life (average 15 years) and 14% would risk a 1/1000 chance of death to receive a treatment that would make them symptom-free [27].

The etiology of IBS is not completely understood, but several factors are believed to play a pivotal role in the pathophysiology of IBS. These factors are genetics, diet, gut microbiota, gut endocrine cells, and low-grade inflammation [1]. Studies of family history and family cluster, as well as twin studies, provide strong evidence that IBS is hereditary [28,29,30,31,32,33,34,35]. However, the gene responsible for IBS has not been determined yet [1,36]. Low-grade inflammation occurs in some IBS patients, but far from all [1]. Diet, gut microbiota, and gut endocrine cells interact with each other in a way that affect IBS symptoms. The present review is an attempt to clarify this interaction and the possible implications for everyday clinical work.

## 2. Diet in IBS

Patients with IBS attribute their symptoms to specific food items such as milk and milk products, wheat products, cabbage, onion, peas/beans, hot spices, and fried food [37,38,39,40,41]. Despite the selective choice of food by IBS patients, the intake of calories, carbohydrates, proteins, and fats by IBS patients is similar to community controls [38,42,43]. There was no difference either in the number of meals or meal patterns between IBS patients and community controls [38]. However, the diets of IBS patients are lower in β-carotene, retinol, riboflavin, calcium, magnesium, and phosphorous [38]. Moreover, IBS patients avoid certain food items, some of which belong to the fermentable oligo-, di-, monosaccharides, and polyols (FODMAPs) group, but they have a high consumption of other food items that are rich in FODMAPs [38]. They also consume more food with probiotic supplements and avoid fewer food sources that are important to their health [38].

The influence of diet on IBS symptoms was previously explained by food allergy/intolerance, and poorly absorbed carbohydrates and fiber [44]. There is no evidence that food allergy/intolerance is involved in IBS, but it is generally accepted that poorly absorbed carbohydrates and fiber play an important role in the development of IBS symptoms [44,45]. The intake of a low FODMAP-diet improves both symptoms and quality of life in IBS patients (Table 1) [37,44,46,47]. However, only 50–70% of IBS patients have effect of low FODMAP-diet [48,49,50]. Moreover, a low FODMAP-diet is expensive and hard to maintain over a long time and changes the intestinal microbiota negatively [49,51,52,53]. Furthermore, the intake of low FODMAP-diet over a long time may cause deficiencies in vitamins, minerals, and naturally occurring antioxidants [52,53]. A NICE (National Institute for Health and Care Excellence)-modified diet (Table 2) has the same effect as a low FODMAP-diet, is easy to maintain, and does not have the hazards seen with the FODMAP-reduced diet [49,50]. The NICE-modified diet is now the diet first recommended to patients with IBS [54,55]. In our clinic, we use a slightly modified NICE diet, as recommended by the British Dietetic Association [37,38]. In this diet, the patients are asked to have regular meals, to replace wheat products with spelt products, to reduce their intake of fatty food, onions, cabbage, and beans, to avoid soft drinks and carbonated beverages, chewing gum, and sweeteners that end with -ol, and to regularly intake of psyllium husk fibers. The British Dietetic Association also recommends reducing coffee drinking, and avoiding spicy foods and alcohol [37,38]. However, these three food items’ association to IBS needs more clarification.

Caffeine is not known to affect IBS symptoms. However, a large number of IBS patients suffer from reflux esophagitis [4], and reducing their coffee intake would improve their reflux symptoms.

IBS patients are known to consume less alcohol than the normal population, and 12% of them avoid alcoholic beverages [38,56,57,58]. Chronic alcohol consumption affects gastrointestinal motility, damages the gut mucosal, impairs nutrient absorption, and causes inflammation [59,60,61,62,63,64]. The mechanism by which alcohol affects gastrointestinal motility is thought to be by inhibiting nitric oxide pathways [59,60,61,62,63]. Whereas the previously mentioned effects of alcohol on the gastrointestinal tract have been detected in chronic alcohol abuse, the effects of moderate social consumption of alcohol on gastrointestinal tract functions are not known.

Whereas some studies have shown that drinking alcoholic beverages induces symptoms in IBS patients [56,65,66], others have not [67]. These contradictory results could be explained by the observation that moderate and light drinking are not associated with IBS symptoms, but binge drinking is [68]. Moreover, there is marked individual variation in alcohol drinking being a trigger of IBS symptoms [68]. The recommendation of the British Dietetic Association is that each individual should assess the relation between alcohol intake and symptom developments in order to determine whether a reduction is necessary. This recommendation works well in the clinical setting.

Capsaicin is the major component in spicy food with hot peppers. Capsaicin accelerates gastrointestinal transit through TRPV receptors, hence causing abdominal pain [69]. Several studies have shown that spicy food induces the onset of IBS symptoms [56,65,66,67,70]. It has been shown, however, that whereas an occasional ingestion of chili increases abdominal pain/discomfort [70,71], chronic intake of chili decreases abdominal pain and bloating in IBS patients [72,73]. This effect seems to be caused by desensitization effects of capsaicin ingestion on TRPV1 receptors [74]. Asians consume 2.5–8g/person of chili daily, which is 10–300 times higher than the intake of Europeans [75,76,77]. This probably can explain why Asian patients with IBS have less abdominal pain and the alteration in bowel habits is much less prominent in Asian IBS patients than in Western patients [78,79,80,81].

## 3. Gut Microbiota

It has been estimated that more than 10^14^ microorganisms are harbored in the human gut [82]. The gut is inhabited by 12 different bacteria phyla, comprising 2172 species. Most gut bacteria belongs to the Proteobacteria, Firmicutes, Actinobacteria, and Bacteroidetes phyla [83]. However, anaerobic Firmicutes and Bacteroidetes dominate the bacterial population in the gut of healthy adults, with a few members from of the Proteobacteria and Actinobacteria phyla [83,84]. A low microbial diversity in the gut (dysbiosis) is associated with several diseases [85,86].

Bacterial composition in healthy subjects is determined by genetics and environmental factors [82,86]. Genetics explains only 5–10% of the bacterial variability between individuals, which emphasizes the importance of environmental factors [86]. Among these environmental factors are diet, the frequency of antibiotic treatment, treatment with certain non-antibiotic drugs, geographical location, surgery, smoking, and depression [82,86,87].

The gut bacterial composition in IBS patients differs from that of healthy subjects [86,88,89,90]. IBS patients have a lower abundance of *Erysipelotrichaceae* and *Ruminococcaceae*, butyrate-producing bacteria, than healthy controls. Whereas *Methanobacterialles*, methane-producing bacteria, are more abundant in IBS-C, they are less abundant in IBS-D than in healthy individuals [91,92]. Moreover, IBS patients have been found to exhibit an increase in the abundance of bacteria belonging to Proteobacteria, *Veillonella*, and Firmicutes, such as *Lactobacillus* and *Ruminococcus,* and a decrease in the abundance of *Bifidobacterium*, *Faecalibacterium*, *Erysipelotrichaceae*, and methanogens, as compared with healthy individuals in the community [91,92]. Furthermore, IBS patients have a lower diversity of gut bacteria (dysbiosis) than healthy subjects [86,88,89,90,91,92,93]. Patients with IBS who did not respond to low-FODMAP or NICE-modified diets have been found to suffer from a severe dysbiosis [58].

Changes in the intestinal microbiota in experimental animals causes gastrointestinal dysmotility, visceral hypersensitivity, altered intestinal permeability, and altered behavior [93]. All of these abnormalities are similar to those encountered in IBS. There is a growing body of evidence showing that intestinal microbiota not only explain the abdominal symptoms of IBS, but also the psychiatric co-morbidity occurring in a considerable number of IBS patients [93]. Although altered gastrointestinal microbiota and dysbiosis in patients with IBS are documented, the microbial signature characterizing these patients is not known yet [93].

## 4. Gut Hormones

**Figure 1:**
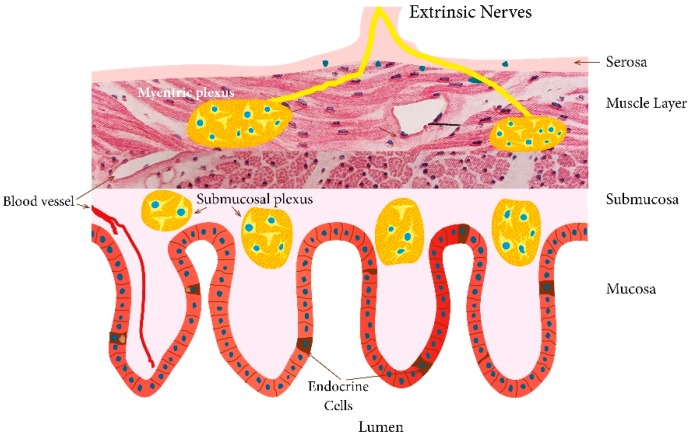
Schematic illustration of the gut endocrine cells. The endocrine cells are scattered among the epithelial cells of the mucosa facing the gut lumen. These cells secret at least 14 different hormones that regulate gut motility, secretion, absorption, visceral sensitivity, local immune defense, cell proliferation, and appetite. These hormones also interact and integrate with the enteric, autonomic, and central nervous systems.

**Figure 2:**
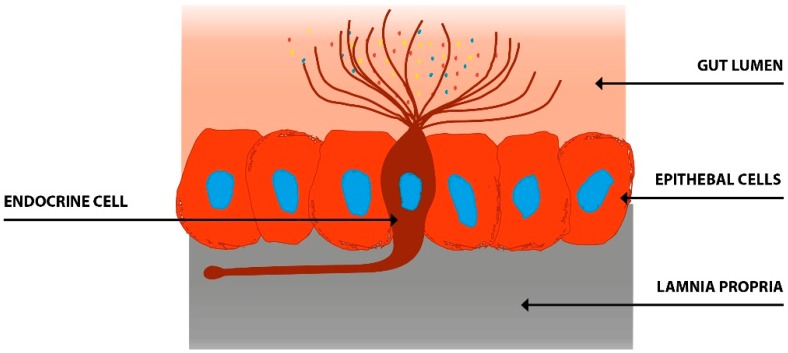
The gut endocrine cells have specialized microvilli that project into the gut lumen and act as sensors for the gut contents (mostly for nutrients). They respond to luminal content by releasing their hormones into the lamina propria. These hormones act locally on nearby structures (paracrine mode of action) or enter the blood stream and act on more distant structures (endocrine mode of action).

There are at least 14 different gut hormones secreted by endocrine cells scattered between the epithelial cells facing the gut lumen [19,94] (Figure 1). These cells are localized to the stomach and the small and large intestine [19]. The different types of gut endocrine cells, their functions, and modes of action have been described in details previously [3,37,94,95,96,97,98,99,100,101,102,103,104,105,106,107,108,109]. These cells have specialized microvilli that project into the lumen and function as sensors for the gut contents (mostly for nutrients), and respond to luminal stimuli by releasing their hormones into the lamina propria (Figure 2) [110,111,112,113,114,115,116,117,118,119,120,121,122]. Different gut hormones are released from the gut endocrine cells depending on the gut intraluminal contents and the proportions of carbohydrates, proteins, and fats [19,94]. Thus, carbohydrate-rich luminal contents stimulate the release of gastric inhibitory peptide (GIP) and enteroglucagon, protein-rich luminal contents cause the release of peptide YY (PYY), pancreatic polypeptide (PP), neuropeptide Y (NPY), motilin, ghrelin, and cholecystokinin (CCK), and fat-rich luminal contents result in the release of neurotensin, enteroglucagon, galanin, motilin, ghrelin, and CCK. These hormones act locally on nearby structures (paracrine mode of action) or by entering the circulating blood and reaching distant targets (endocrine mode of action) [123]. These hormones interact and integrate with each other, with the enteric nervous system, the autonomic nervous system, and the central nervous system [3,94,98,124]. Gut hormones regulate several functions of the gastrointestinal tract, including visceral sensation, motility, secretion, absorption, local immune defense, cell proliferation, and food intake [3,96,108,109,124].

**Figure 3:**
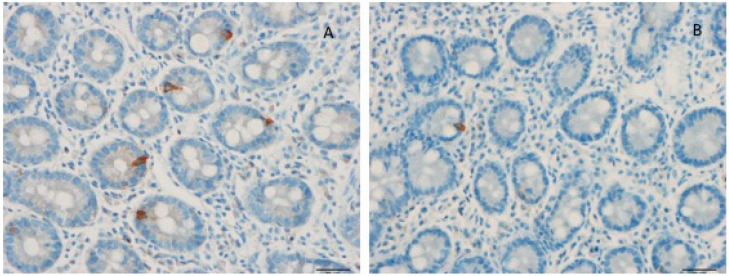
Duodenal cholecystokinin (CCK) cells of a healthy subject (**A**) and of a patient with irritable bowel syndrome (IBS) (**B**). Patients with IBS have a low density of CCK cells.

**Figure 4:**
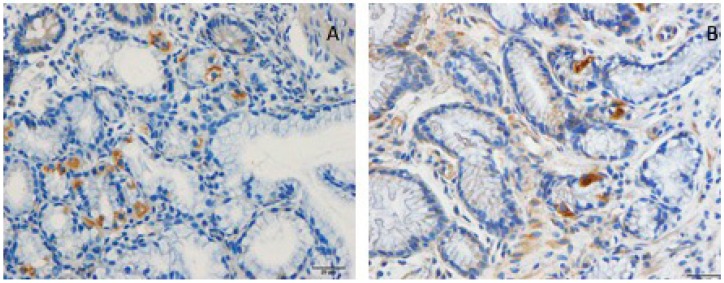
Musashi 1 cells in the duodenum of a healthy subject (**A**) and in the duodenum of a patient with IBS (**B**). Musashi 1 is a marker for stem cells and their early progenitors.

Several abnormalities in different endocrine cell types of the stomach and the small and large intestine have been described in IBS patients (Figure 3) [125,126,127,128,129,130,131,132,133,134,135,136]. Generally, IBS patients have a lower gut endocrine cell density than healthy subjects [94]. A low density of gut endocrine cells occurs in patients with congenital malabsorptive diarrhea, in small intestine allograft rejection, and in NEUROG3-knockout mice [137,138,139]. The low density of gut endocrine cells in these conditions is accompanied by a reduction in the number of gut neurogenin 3 cells. [137,138,139]. Neurogenin 3 is a marker for early intestinal endocrine cell progenitors originating from stem cells, which are located at the base of the crypts [138,140,141,142,143,144,145]. In the small and large intestines of patients with IBS, the cell density of Musashi 1 and neurogenin 3 are lower than that of healthy subjects (Figure 4) [135,146,147,148]. Musashi 1 is a marker for intestinal stem cells and their early progeny [138,140,141,142,143,144,145]. 

Accordingly, it is probable that a low number of these in the stem cells and enteroendocrine cells progenitors might be responsible for the low density of gut endocrine cells [149].

## 5. Interaction between Diet, Microbiota, and Endocrine Cells in the Guts of Patients with IBS

**Figure 5:**
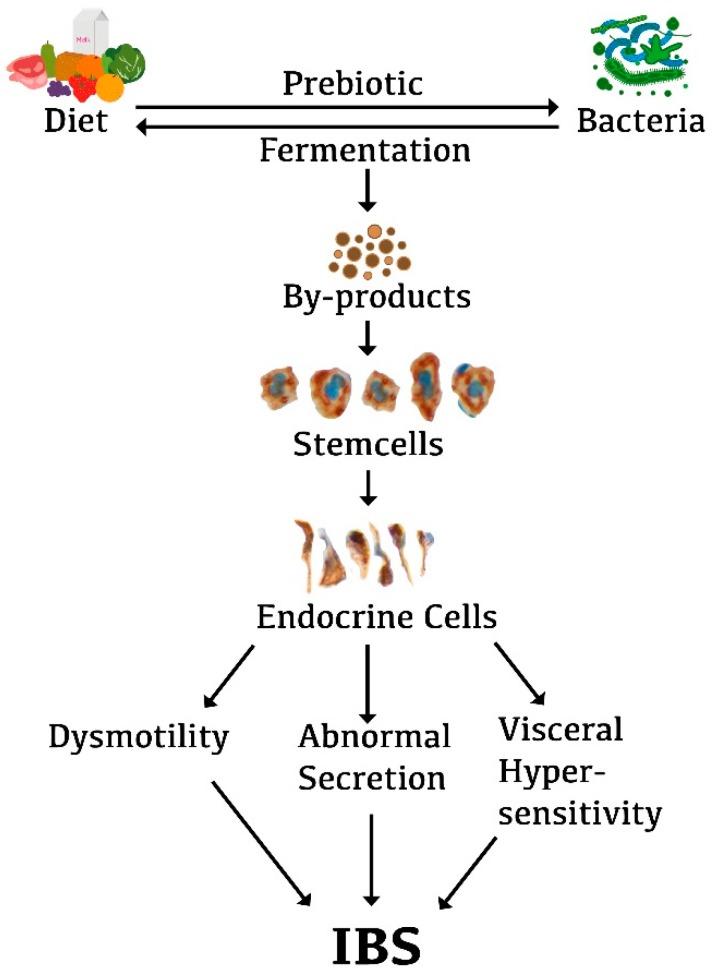
Schematic illustration of the possible role of the interaction of diet, gut microbiota, and gut endocrine cells in the pathophysiology of IBS. The foods we ingest act as prebiotics that favor the growth of a certain type of bacteria. These bacteria in turn ferment the diet, resulting in by-products. These by-products may act on the stem cells in a way that reduces their number. This in turn would result in a low density of gut endocrine cells. The low density of gut endocrine cells gives rise to the gut dysmotility, visceral hypersensitivity, and abnormal gut secretion that are seen in IBS patients.

The food that we ingest acts as a substrate (prebiotics) for intestinal bacteria. Our choice of different food items determines our intestinal bacterial profile. On the other hand, intestinal bacteria ferment the undigested remains of the food items into methane and hydrogen gases, as well as short chain fatty acids [92] (Figure 5). Fructans and galactans are substrates for several bacteria and a low-FODMAP diet appears to induce unfavorable changes in the intestinal bacterial profile of IBS patients [150,151]. Patients with IBS adhering to a low-FODMAP diet develop a lower abundance of fecal *Bifidobacteria* as compared with healthy subjects/before intervention [150,151]. Moreover, the absolute and relative numbers of butyrate-producing bacteria have been reported to be reduced following a low-FODMAP diet intervention [150,151].

Patients with IBS have gut dysmotility, visceral hypersensitivity, and abnormal secretion [146]. The hormones secreted from gut endocrine cells regulate gut motility, visceral sensitivity, and secretion [39]. The abnormalities in the gut endocrine cells seem to explain the gut dysmotility, visceral hypersensitivity, and abnormal secretion seen in IBS patients [148]. It is believed that the abnormalities in the gut hormones play a major role in the pathophysiology of IBS [148,149].

The gut hormones, as mentioned previously, sense the luminal content of the gut and release their hormones accordingly. Gut luminal contents rich in proteins cause the release of certain hormones, while those rich in carbohydrates or fats cause the release of other hormones.

The composition of the diet, with different proportions of carbohydrates, proteins, and fats, is a trigger for the release of different gut hormones into the lamina propria [40,46].

Diet seems to also interact with gut endocrine cells in more complicated ways. It has been shown recently that a change in diet from a common Norwegian diet to a low-FODMAP diet results in a change in the density of gastrointestinal cells towards the levels of healthy subjects [152,153,154,155,156,157,158]. The effect of a NICE-modified diet is not known. Furthermore, fecal microbiota transplantation (FMT) changes the density of small and large intestine endocrine cells in patients with IBS [159]. These changes in gut endocrine cells caused by a low-FODMAP diet and FMT was accompanied with an improvement in symptoms and quality of life [159].

One may speculate that diet acts as prebiotic favoring the growth of certain bacteria. These bacteria in turn ferment the diet, which results in by-products. These by-products act on gut stem cells, causing low differentiation into endocrine cells. The low density of gut endocrine cells and the subsequent low levels of certain hormones give rise to gut dysmotility, visceral hypersensitivity, and abnormal secretion (Figure 5).

## 6. Conclusions

Several endocrine cell types are scattered among the epithelial cells of the gut mucosa and secret at least 14 different hormones. These hormones regulate many functions of the gut. Some of these cells response to protein-rich luminal contents by releasing their hormones. Others response to carbohydrates or fat-rich luminal contents. Thus, the proportions of proteins, carbohydrates, and fats in the diet, and consequently in the gut lumen, determine which hormones are released.

The density of gut endocrine cells is lower in IBS patients than healthy subjects, probably because of a low density of stem cells and low differentiation of these cells into endocrine cells. It is believed that this abnormality plays a major role in the pathophysiology of IBS. A low FODMAP intake and FMT improve symptoms and quality of life and restore the density of the gut endocrine cells. An interaction between diet, gut microbiota, and gut endocrine cells appears to play an important role in the pathophysiology of IBS.

This interaction should be kept in mind when managing IBS patients in clinic. Patients who do not respond well to diet management should be considered as candidates for gut microbiota alteration through FMT.

## Figures and Tables

**Table 1:** Food items rich in fermentable oligo-, di-, monosaccharides, and polyols (FODMAPs).

Vegetables	Fruits	Others
Onions, garlic, the white portion of leeks and spring onions, cabbage, spring onions, mushrooms, beans, red kidney beans, Brussels sprouts, sugar peas, asparagus, lentils, beets, artichoke, fennel, peas, sugar peas, cauliflower	Apples, pears, peach, mango, watermelon, dried fruit, fruit juice, canned fruit in natural juice, nashiphary, apricot, longan, cherry, lychee, nectarine, plum	Wheat, barley, rye, bread, pasta, couscous, biscuits, cakes
		Milk and dairy products: cheese, yogurt, soy milk, cream
		Sweeteners containing fructose (for example, corn syrup)
		Sweeteners: sorbitol, mannitol, xylitol, isomalt, maltitol, and other sweeteners with names ending in “ol”

**Table 2:** The food items that should be avoided in a National Institute for Health and Care Excellence (NICE)-modified diet.

Vegetables	Fruits	Others
Onions, garlic, beans, peas, artichoke, cabbage	Watermelon	Wheat flour and wheat-based products Milk and dairy products Sweeteners containing fructose (for example, corn syrup) Sweeteners: sorbitol, menthol, xylitol, isomalt, maltitol, and other sweeteners with names ending in “ol” Carbonated drinks (soft drinks), coffee, beer
